# Community based cross sectional study of podoconiosis and associated factors in Dano district, Central Ethiopia

**DOI:** 10.1371/journal.pntd.0007050

**Published:** 2019-01-28

**Authors:** Feven Dejene, Hailu Merga, Henok Asefa

**Affiliations:** 1 Public Health Emergency management, Ethiopian Public Health Institute, Addis Ababa, Ethiopia; 2 Department of Epidemiology, Institute of Health, Jimma University, Jimma, Ethiopia; University of California San Diego School of Medicine, UNITED STATES

## Abstract

**Background:**

Podoconiosis, affects lower limb, is an entirely preventable non-communicable tropical disease common in low income countries. Globally it is estimated that there are 4 million peoples with podoconiosis and nationally it is estimated that there are 1.56 million cases of podoconiosis. Even though nationwide mapping has been conducted including the current district under investigation, there are no studies conducted to identify factors associated with podoconiosis in the district. Hence, this study was aimed to determine the prevalence of podoconiosis and associated factors in the west Shewa of Dano district community.

**Method:**

A community based cross sectional study was conducted from March 1 to 26, 2018. Seven kebeles out of the total of twenty-three kebeles found in the district were selected randomly. The total sample size was allocated by probability proportional to size to each kebele based on the number of households they had. Then, systematic random sampling was employed to select 652 study participants from the seven kebeles. Data was collected using interviewer administered structured questionnaire and observation. In addition, a blood sample was collected from the study subjects who had leg swelling for ruling out lymphedema due to lymphatic filarasis by using Immunochromatographic test card. Podoconiosis case was defined as bilateral but asymmetric swelling which develop first in the foot often confined to the lower leg and negative result for immune-chromatographic test (ICT card). The prevalence of podoconiosis was determined and multiple logistic regression model was fitted using SPSS version 23 to identify factors associated with podoconiosis.

**Result:**

The prevalence of podoconiosis in Dano district was found to be 6.3% (95%CI: 5.8, 6.8). Age at first shoe wearing (AOR = 1.08,95% CI = 1.06–1.11), washing practice of feet by water only (AOR = 3.68, 95% CI = 1.47–9.24) and not wearing shoe daily (AOR = 9.32, 95% CI = 4.27–20.4) were found to be significantly associated with increased odds of podoconiosis.

**Conclusion:**

This study revealed that there was significant burden of podoconiosis in the study area. Age at first shoe wearing, washing practice and frequency of shoe wearing were associated with the development of podoconiosis disease. Modalities to enhance the shoe wearing behaviour of the communities should be planned by high level decision makers working in the area of Health. Moreover, collaboration between local government and non-government stakeholders, and integration with existing programs addressing foot hygiene which involves washing feet with soap and water needs to be addressed.

## Author summary

Podoconiosis (endemic non-filarial elephantiasis) is one of a disabling and stigmatizing neglected tropical disease (NTD) which affects the lower limb found mostly in low income countries. Even though there were mostly prevalence studies and disease mapping, there was no report, especially on factors associated with the disease, from the study area. Hence, community based cross sectional study was conducted to determine its prevalence and factors associated with it in the west Shewa Zone of Dano district community. Data were collected from a total of 652 randomly selected peoples using interviewer administered structured questionnaire and observation. In addition, a blood sample was collected from the study subjects who had leg swelling for ruling out lymphedema due to lymphatic filarasis by using Immunochromatographic test card. Podoconiosis case was defined as bilateral but asymmetric swelling which develop first in the foot often confined to the lower leg and negative result for immune-chromatographic test (ICT card). Accordingly, the prevalence of podoconiosis in the district was found to be 6.3%. Age at first shoe wearing, washing practice of feet by water only and not wearing shoe daily were found to be the risk factors for the development of podoconiosis. Hence, this study revealed that there was significant burden of the disease in the study area. Thus, collaboration between local government and non-government stakeholders, and integration with existing programs addressing foot hygiene which involves washing feet with soap and water needs to be addressed.

## Introduction

Neglected tropical diseases (NTDs) are prevalent in many tropical and sub-tropical developing countries where poverty is rampant. Evidence showed that one–sixth of the world’s population, mostly in developing countries, are infected with one or more of the NTDs. The World Health Organization (WHO) has identified seventeen NTDs for control and elimination at the global level [1,2] and among these diseases, eight were identified, including podoconiosis as priority in Ethiopia with a range of endemicity across the regions [3]. Historically, podoconiosis was widespread in Europe and North Africa too, but disappeared when the practice of wearing a shoe became common [4].

Podoconiosis (an endemic non-filarial elephantiasis), an entirely preventable non-communicable tropical disease, is one of a disabling and stigmatizing NTD, which affects the lower limb is found mostly in low income countries [5–7]. It has been described in more than ten countries across tropical Africa which bear the highest disease burden and has also been reported in tropical areas of the central America and southeast Asia [8,9]. It is the health problem in more than ten African countries, including Tanzania, Uganda, Kenya, Cameroon and Ethiopia [10–14].

In Ethiopia, the soil responsible for the disease is estimated to cover 24% of the surface area of the country on which an estimated 43.8% of the surface area of the population lives. And the national average prevalence was 4% with the highest prevalence in SNNPR (8.3%) followed by Oromia (4%) and Amhara (3.9%) regional states. Nationally it is estimated that there are 1.56 million cases of podoconiosis and there were 345 districts with the prevalence of disease greater than 1% [15]. Study revealed that up to 64% affected individuals are most economically active age groups and it is estimated that podoconiosis patients lose 45% of total working days per year [16]. Stigmatization against podoconiosis patients is common, with patients being excluded from school, churches, mosques and barred from marriage with unaffected individuals. Podoconiosis has severe health, social and economic consequences. It is modern, the silent public health disaster made up of hundreds of thousands of men, women and children sufferings the sham of skin disease [17]. It causes painful swelling and deformity of the lower legs with acute, painful inflammatory events known as acute adenolmphangioadenitis [18]. As a result, Ethiopia has planned to eliminate podoconiosis by 2030 [15]. Study found that, in addition to few socio-demographic factors, leg washing practice and regular travel by barefoot are predictors of podoconiosis [19]. Some studies have shown that females are at greater risk of developing podoconiosis, while other studies have showed no sex differences [20–22]. In Ethiopia clinical, parasitological, and serological examinations are used to diagnose podoconiosis. Mostly day time finger-prick blood sample is obtained from the patients with elephantiasis for serological examination and Immunochromatographic test (ICT), the WHO recommended Gold standard test, is commonly used. Even though there were mostly prevalence studies and disease mapping, there was no report, especially on factors associated with the disease, from the study area. Hence, this study was aimed at determining the prevalence of podoconiosis and associated factors in the community of Dano district in West Shewa of Oromia regional state.

## Methods and materials

**Fig 1:**
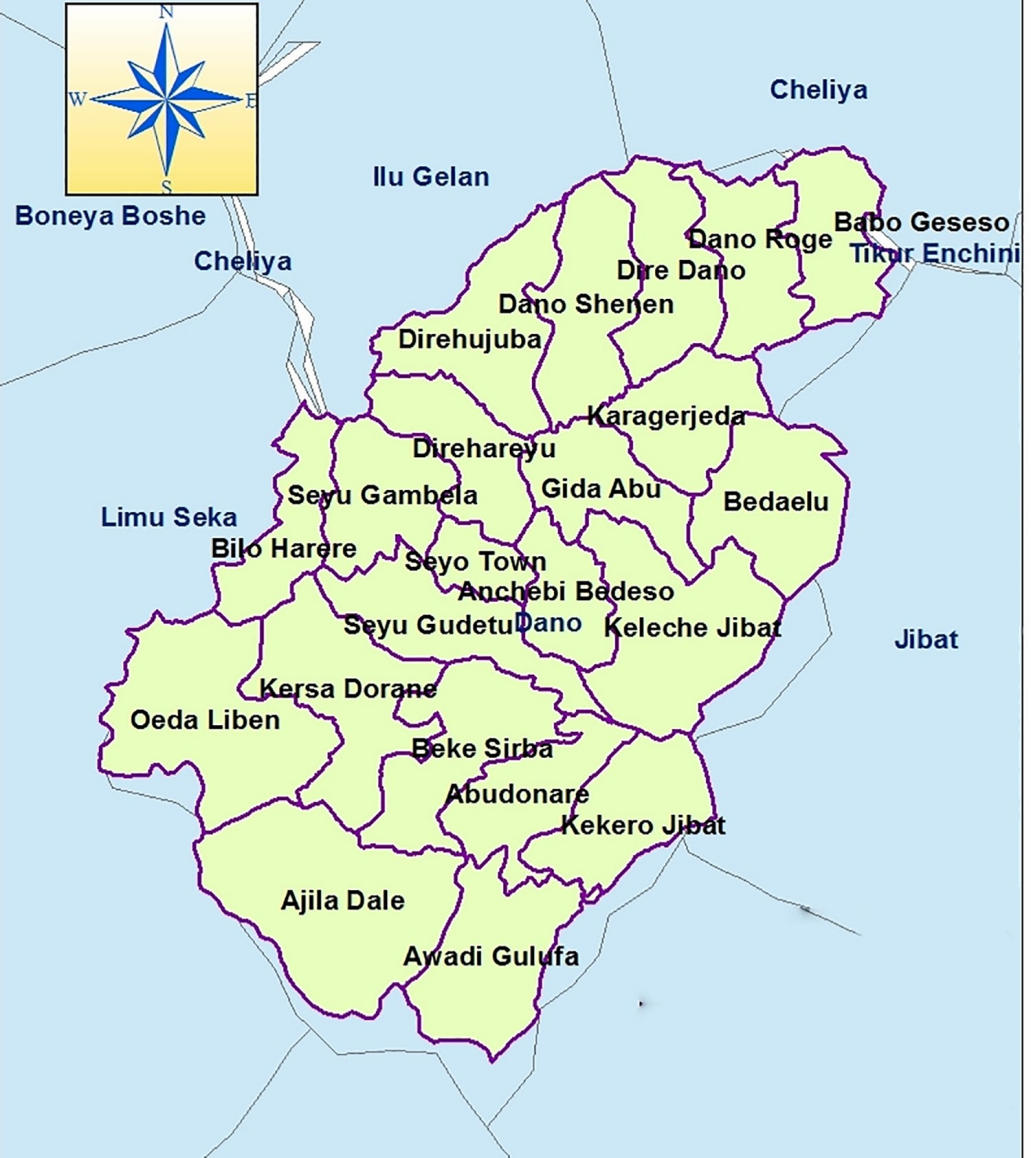
Map of Dano district to assess the magnitude of podoconiosis and its associated factors.

A Community based cross sectional study was conducted in Dano district from March 01 to 28, 2018. This district is one of the 22 districts in West Shewa zone, Oromia regional state. Dano district, which is located in the west of the capital, Addis Ababa, is bordered on the southwest by the Jimma zone, on the north by cheliya district, and on the southeast by Nano district (Fig 1). The total population for this district were 97,243 of whom 48,593 were men and 48,650 were women [23].

The source population was all individuals living in the district whose age was greater than 15 years while the study population was all individuals included in the study whose ages were greater than 15 years living in selected kebeles (the smallest administrative unit in Ethiopia). Study participant who had lost their both legs due to different health problems and accident were excluded from the study.

### Sample size and sampling

The sample size was calculated using double population proportion formula using statcal program of Epi-info tool by considering confidence level 95%, Power of 80%, 5% precision, 9% proportion of illiterate who had podoconiosis and 90.9% proportion of illiterate who were free from podoconiosis with its AOR 10.1 from previous study [19]. Then, by using design effect of 2 and adding 10% non-response rate, the final sample size for this study was 652. Seven kebeles out of the total of twenty-three kebeles found in the district was selected randomly. The total sample size was allocated by probability proportional to size to each kebele based on the number of households they had. List of the households which was obtained from health extension workers was served as sampling frame for selection of households. Systematic random sampling technique was employed to select 652 study participants from the seven kebeles found in the district. The sampling interval for each kebele was calculated by dividing the total number of households in each kebele to the total number of households selected from each kebele. A random starting point was fixed for each kebele to select the first household by using each kebele center as starting point and pen was pinned to identify the beginning direction. Then, every k^th^ household was selected until the required sample size was obtained from each selected kebele. In case more than one eligible study subjects found in the household, one study subject was selected randomly by lottery method. Similarly, in case there was no eligible subject in the selected household, the next immediate neighbor's household with eligible study subject was included in the study.

### Data collection tool and procedures

Data was collected using interviewer administered structured questionnaire and observation checklist. The questionnaire includes socio-demographic factors, behavioral factors, genetic factor and housing conditions. A questionnaire was adapted and prepared from related literature with modification to local context [10,12,18–20,24–26] (S2 Text). Six diploma nurses for data collection, three laboratory technologists for blood collection and two Public Health officers for supervision were recruited from Dano health center (nearby health center) and trained in data collection. The Blood sample was collected for study subjects who had leg swelling for ruling out lymphedema due to lymphatic filariasis by using Immunochromatographic test (ICT) (Binax, Inc., Scarborough, ME, USA). The control lines developed in the ICT test indicated that the cards were valid. Previous studies showed that the sensitivity and specificity of the ICT filarasis card test were 100% when compared with Knott’s concentration and counting chamber methods and 85–100% and 96–100%, respectively when compared with three other tests. The ICT card test is rapid, relatively simple to use in the field settings, and can be used with day or night blood samples. It has been regarded by the WHO as the “Gold Standard” for diagnosis of LF [27]. Podoconiosis case was defined as bilateral but asymmetric swelling which develop first in the foot often confined to the lower leg and negative result for immune-chromatographic test (ICT card).

To maintain the quality of data, standardized and data collection tools were used. The questionnaire was prepared in English and then translated into regional language Afan Oromo then back to English to ensure consistency. The questionnaire was pre-tested in Bako district (nearby district) on 5% of the sample size (33 households). Three days training was given for data collectors and the supervisor. Daily supervision was carried out to check the completeness of the questionnaire. Standard diagnostic material was used and standard operating procedure was followed by trained laboratory technologists. The data were entered twice into Epi Data software to further minimize data entry error. In addition, at the end of data entry, data cleaning was performed to check missed values and outliers. Errors identified during data collection were corrected accordingly at the field and those errors occurred during or after data entry was corrected by revising the original questionnaire.

Family history of podoconiosis was measured as having one or more affected close relatives (parent or grandparent) by podoconiosis and frequency of feet washing was measured as respondents`who wash their feet at least once per day. Similarly, frequency of shoe wearing was defined as respondents`who wear shoe daily.

### Analysis

STROBE checklist was used to analyze and report data [28] (S1 Text). Data entering, coding and clearing were done by Epi Data version 3.1 to minimize data entry error and exported to Statistical Package for Social Sciences (SPSS) version 23 (IBM SPSS Statistics for Macintosh, Version 23.0 Armonk, NY) for analysis. Descriptive analysis was done to describe variables involved in study. Uni-variate and bi-variate analyses were done to identify candidate variables for the final model (with p-value ≤0.25) and Multivariable (with p-value < 0.05) logistic regression analysis was performed to identify independent factors associated with podoconiosis. The degree of association was assessed by using Adjusted Odds Ratio (AOR) with 95%-CI. P-values < 0.05 were used as statistical criterion of significance. The model goodness of fit was tested by Hosmer and Lemshow goodness fit test.

### Ethics statement

Ethical clearance was approved and obtained from Ethical review board of Institute of Health, Jimma university (JUIRB/THRPGD/14/2018) and formal letter was obtained from Oromia regional Health bureau. Then, the letter was submitted to Dano district Health office. Before interview and taking sample, written informed consent was obtained from each respondent or their parents or legal guardian after explaining them the objective and significance of the study. To maintain confidentiality, names of the informant were not written across the study. Informant’s involvement in the study were on voluntary basis and those who wish to quit their participation at any stage was informed to do so without any restriction. In individuals aged below 18 years, informed consent was obtained and interview was conducted to the parents or legal guardian.

## Results

### Background characteristics of respondents

Out of 652 sample size calculated, a total of 638 participants participated in the study giving a response rate of 97.8%. From the total participants, 395 (61.9%) were females. The mean age (±SD) of the study participants was 35.75 (±12.84) years. One third (30.4%) of the study participants had no formal education and about half (47.1%) of them were Farmers (Table 1).

**Table 1:** Socio-demographic characteristic of study participants in Dano district, West shewa Zone, Oromia, Ethiopia, 2018.

Variable	Frequency	Percent %
**Age**		
15–24	113	17.7
25–34	201	31.5
35–44	145	22.7
45–54	124	19.4
>55	55	8.6
**Sex**		
Female	400	62.7
Male	238	37.3
**Education**		
No formal education	194	30.4
Able to read and write	164	25.7
Grade 1–8	181	28.4
Grade 9–12	51	8.0
More than secondary	48	7.5
**Occupation**		
Farmer	301	47.1
House wife	86	13.5
Merchant	71	11.1
Student	70	11.0
Government employee	33	5.2
Non-government employee	33	5.2
Non-government employee	32	5.0
Daily labor	12	12
Other*		
**Religion**		
Orthodox	304	47.6
Protestant	256	40.1
Muslim	72	11.3
Others	6	0.94
**Ethnicity**		
Oromo	600	94
Others**	38	6
**Marital status**		
Married	375	58.8
Never married	125	19.6
Living together	60	9.4
Widowed	45	7.1
Divorced	33	5.2
**Monthly income**		
<500	447	70.1
≥501	191	29.9

* unemployed,

**Amhara, Gurage and Tigre

### Prevalence of podoconiosis

Of the 49 lymphedema cases, 9 cases who were diagnosed lymphatic filariasis were found ICT positive and had swelling which started from high up in the leg also symmetric swelling in which confined to thigh, but there was no groin involvement. The prevalence of podoconiosis in this study was found to be 6.3% (95%CI: 5.8, 6.8). The proportion of podoconiosis among female were 62.5%. The higher prevalence of podoconiosis, 2.2%, was observed among people age groups 45–54 years whereas the lower prevalence, 0.16%, was observed among both age groups 15–24 years and age 65 years and above (Table 2). Majority (50%) of the podoconiosis were in second stage of the disease, 30% were in stage three, 10% were in stage one, 7.5% were in stage five and 2.5% were at stage four of the disease.

**Table 2:** Age and sex distribution of respondents who had podoconioisis in Dano district, 2018.

Variables	Number of individuals screened	Number of podoconiosis patients	Prevalence
**Sex**			
Female	400	25	3.92
Male	238	15	2.35
**Age**			
15–24	114	1	0.16
25–34	201	8	1.25
35–44	145	7	1.1
45–54	122	14	2.2
55–64	35	9	1.4
> = 65	21	1	0.16

The mean age at first shoe wearing (±SD) of the study participants was 13.12±9.37 years. Out of the study participants, only three of them never wore shoe while twenty-eight percent of the them reported to wore shoes for the first time when they were older than 26 years. More than three fourth (86.5%) of the respondents wore shoes at time of interview. Regarding site of shoe wearing, 85.3% and 87.1% of them wore shoe at home and field respectively. Similarly, 87.1%, 97.5%, 98.1% and 94% of the study participants wore shoe during rainy season, at market day, at Sunday and when walking far respectively. From podoconiosis affected participants, 16 (40%) didn`t wear shoe daily. Most (95.5%) of study participants that had travelled for social purposes wore shoe and also 89.3% of them wore shoe during farming. About 91.2% of the study subjects washed their feet by using water and soap. Regarding the frequency of feet washing, about two third (64.3%) of the study subjects washed their feet twice per day. During the interview for data collection it was observed that, 591 (92.6%) of participants had clean and intact feet.

Concerning with genetic factors, about half (52.5%) of the podoconiosis patients had mentioned that they had at least one blood relative with similar condition. Most of the participants, 565(88.6%) had their house floor made of mud. Distance from their water source was assessed and accordingly, 98.3% of the respondents mentioned that they had access to water less than 30 minutes.

### Factors associated with podoconiosis

Age (p = 0.001), educational status (p = 0.073), occupation (p = 0.002), age at first shoe wearing (p = 0.001), frequency of shoe wearing (p = 0.001), washing practice (p = 0.001), distance from nearest water source (p = 0.001), regular walking for different social purpose on barefoot (p = 0.01), shoe wearing during farming (p = 0.002) and having family leg swelling history (p = 0.001) were found to be associated with podoconiosis in the bi-variate analysis using p-values ≤ 0.25 cutoffs.

After multivariable logistic regression analysis, age of respondents, frequency of shoe wearing and their legs washing practice were significantly associated with podoconiosis. As participants age of shoe wearing delays by five years, the risk of developing podoconiosis increases by 49% (AOR = 1.08, 95% CI = (1.06–1.11)). Podoconiosis among participants who washed their feet only by water were about 4 times higher as compared to those who washed by water and soap (AOR = 3.68, 95%CI = 1.47–9.24, p = 0.005). The odds of developing podoconiosis among those who didn`t wear shoe daily were nearly 9 times higher compared to those who wore daily (AOR = 9.32, 95% CI = 4.27–20.4, p = 0.001) (Table 3).

**Table 3:** Factors associated with podoconiosis in Dano district, 2018.

Characteristics	Podoconiosis		COR (95%CI)	AOR (95%CI)	P-value
	Yes, N (%)	No, N (%)			
**Age at first shoes wearing**			1.06(1.04–1.07)	**1.08(1.06–1.11)**	0.001*
**Frequency shoe wearing**					
Daily	23(3.6)	68(10.7)	1	1	
Not daily	17(2.7)	503(83.1)	10.5 (5.37–20.73)	**9.32(4.27–20.4)**	0.001*
**Washing practice**					
Water only	11(1.7)	41(6.4)	5.15(2.4–11.053)	**3.68(1.47–9.24)**	0.005*
Water and soap	29(4.5)	557(87.3)	1	1	

COR: Crude Odds Ratio; AOR: Adjusted Odds Ratio;

*indicates statistically significant at p-<0.05

## Discussion

This study showed that, the prevalence of podoconiosis in the community of Dano district was 6.3%. The finding is in line with the study conducted in Midakegn district, Ethiopia 7.4% [20]. The similarity might be due to the study setting as the two districts located in the same zone. Moreover, the highest prevalence in these districts could be due to low level of foot wear use and access to water, along with the environmental suitability for the disease occurrence, namely soil typically rich in clay particles [14]. However, this prevalence is high compared to studies from Soddo zuria, Ethiopia 5.4% [19], Gulliso district, Ethiopia 2.8% [16], Wayu district, Ethiopia 3.05% [26] and Bedele zuria of west Ethiopia 5.6% [22] as well as few African counties like Kenya 3.4% [12] and Cameron 0.5% [13]. This difference might be due to the difference in study setting and design as well as the intervention provision strategy in those different study settings.

The lowest magnitude of the disease was observed in Soddo Zuria (wolaita Zone) is due to the fact that intervention was preformed that provides health education through oral presentation for the community along with free shoes and socks two to three times annually for children [29]. In addition, most study used only physical examination to identify podoconiosis; but at late stage of the disease, the clinical signs mislead with lymphatic filariasis that lead to under estimation of the result [24].

Age at first shoe wearing is one of the factors associated with the development of podoconiosis. Accordingly, as the age at first shoe wearing delays by 5 years, the risk of podoconiosis increases by 49%. This finding is consistent with studies done at Soddo Zuria district, southern Ethiopia [19]. The reason might be delay of shoe wearing lead to predisposition to cumulative contact of uncovered feet to red soil that increases the risk of developing podoconiosis [6].

The current study showed, shoe wearing habit is also associated with the development of podoconiosis. The odds of developing podoconiosis among those who didn`t wear shoe daily were 9 times higher compared to who wore daily. This finding is in line with study done in Kenya [12]. This is because of minute mineral particles enter into the skin due to long-term exposure red clay of uncovered feet. This triggers a provocative reaction in the lymphatic system which causes thickening and subsequent obstruction of lymphatic system [30]. However, a report from Ethiopia showed that shoe wearing was not found to be associated with podoconiosis and explained the reason might be due to the reverse causality, people with the disease tend to start wearing shoes after developing the disease to conceal the swelling or to prevent its progression [14]. The variation between our result and this report is most likely due to differences in sample size, sampling and the study setting- it is nationwide report and the sample size used was too large as compared to our sample size.

Podoconiosis among participants who washed their feet by water only were 4 times higher as compared to those who washed by water and soap. This is consistent with study done in Soddo zuria, Southern Ethiopia [19]. This is because washing with soap removes soil particles from the foot and restores the function of the skin [31].

### Strength and limitation

The study has strength like using ICT diagnostic tests to exclude lymphoedema caused by lymphatic filariasis. The limitation of this study is the possibility of recall bias that might have been introduced due to some questions were difficult to remember because of time. To minimize such bias training on probing techniques has been given to the data collectors and supervisors. The other limitation of this study is lack of published article on the subject to compare our findings with specifically for the factors associated with the disease. Notwithstanding these limitations, we believe that our study has very important findings for strengthening the prevention and control of podoconiosis in the study area and areas with similar set up.

### Conclusion

This study revealed that there was significant burden of podoconiosis in the study area. Age at first shoe wearing, washing practice and frequency of shoe wearing were associated with the development of podoconiosis disease. Modalities to enhance the shoe wearing behavior of the communities should be planned by high level decision makers working in the area of Health. Collaboration between local government and non-government stakeholders, and integration with existing programs addressing foot hygiene which involves washing feet with soap and water needs to be addressed.

## Supporting information

S1 Text Checklist. STROBE checklist for cross sectional studies.
(DOCX)

S2 Text Tool to assess prevalence of podoconiosis and its associated factors. (DOCX)
