# Supplementary material for: Community based cross sectional study of podoconiosis and associated factors in Dano district, Central Ethiopia
Source: PLoS Negl Trop Dis. 2019 Jan 28;13(1):e0007050. doi: 10.1371/journal.pntd.0007050 (PMC6366781; doi:10.1371/journal.pntd.0007050)
Supplement: S1 Text — STROBE checklist for cross sectional studies. (DOCX) [file pntd.0007050.s001.docx]

**STROBE Statement—Checklist (Cross sectional study)**

|  | **Item Number** | **Recommendation** |
| --- | --- | --- |
| **Tile and Abstract** | **1** | 1. Indicate the study’s design with a commonly used term in the title or the abstract   ***- ‘****community based cross sectional study’ included in the title*   1. Provide in the abstract an informative and balanced summary of what was done and what was found   - *see the whole Abstract* |
| **Background/Rationale** | **2** | Explain the scientific background and rationale for the investigation being reported  *-Introduction, Paragraph 2 and 3*  *- “podoconosis has severe health, social and economic consequences. It is modern, the silent public health disaster made up of hundreds of thousands of men, women and children sufferings the sham of skin disease (17). It causes painful swelling and deformity of the lower legs with acute, painful inflammatory events known as acute adenolmphangioadenitis (18). As a result, Ethiopia has planned to eliminate podoconosis by 2020 (15). Even though there are reports from southern Ethiopia, data from other parts of the country including the study area are limited (4)…….”* |
| **Objectives** | **3** | State specific objectives, including any prespecified hypotheses  **-***Introduction, paragraph 3*  ***- “****Hence, this study was aimed at determining the prevalence of podoconosis and associated factors in the community of Dano district…..”* |
| **Methods** | | |
| **Study design** | **4** | Present key elements of study design early in the paper  - *Methods, paragraph 1*  “*A Community based cross sectional study was conducted in Dano district Ethiopia from march 01 to 28, 2018*” |
| **Setting** | **5** | Describe the setting, locations, and relevant dates, including periods of recruitment, exposure, follow-up, and data collection  *- methods, paragraph 1* |
| **Participants** | **6** | Give the eligibility criteria, and the sources and methods of selection of participants  -*methods section, paragraph 2 and under sample size and sampling title*  *“The source population was all individuals living in the district whose age was greater than 15 years while the study population was all individuals whose age was greater than 15 years living in selected kebeles (smallest administrative unit in Ethiopia). Study participant who had lost their both legs due to different health problems and accident were excluded from the study* ” |
| **Variables** | **7** | Clearly define all outcomes, exposures, predictors, potential confounders, and effect modifiers. Give diagnostic criteria, if applicable  -*methods, under data collection tool and producers, and analysis sections* |
| **Data sources/ measurement** | **8** | For each variable of interest, give sources of data and details of methods of assessment (measurement). Describe comparability of assessment methods if there is more than one group  -*methods section*  *“Data was collected using interviewer administered structured questionnaire and observation checklist. The questionnaire includes social-demographic factors, behavioral factors, genetic factor and housing conditions. A questionnaire was adapted and prepared from related literature with modification to local context (10,12,18–20,24–26)…….”* |
| **Bias** | **9** | Describe any efforts to address potential sources of bias  -Analysis section of methods part  “...*Multivariable (with p-value < 0.05) logistic regression analysis was performed to identify independent factors associated with podoconosis using SPSS. The degree of association was assessed by using Adjusted Odds Ratio (AOR) with 95%-CI. P -value of < 0.05 were used as statistical criterion of significance. The model goodness of fit was tested by Hosmer and Lemshow goodness fit test”* |
| **Study size** | **10** | Explain how the study size was arrived at  “*The sample size was calculated using double population proportion formula using stat cal program of Epi-info tool by considering confidence level 95%, Power of 80%, 9% proportion of illiterate who had podoconiosis and 90.9% proportion of illiterate who were free from podoconiosis with its AOR 10.1. Then, by using design effect of 2 and adding 10 % non-response rate the final sample size for this study was 652*” |
| **Quantitative variables** | **11** | Explain how quantitative variables were handled in the analyses. If applicable, describe which groupings were chosen and why  *-analysis section of methods part*  *”Data entering, coding and clearing were done by Epi Data version 3.1 to minimize data entry error and exported to SPSS version 23 for analysis. Descriptive analysis was done to describe variables involved in study. The prevalence of podoconosis was determined. Uni-variate, Bi-variate were done….”* |
| **Statistical methods** | **12** | 1. Describe all statistical methods, including those used to control for confounding   *-statistical analysis section*  *“Multivariable (with p-value < 0.05) logistic regression analysis was performed to identify independent factors associated with podoconosis using SPSS. The degree of association was assessed by using Adjusted Odds Ratio (AOR) with 95%-CI. P -value of < 0.05 were used as statistical criterion of significance. The model goodness of fit was tested by Hosmer and Lemshow goodness fit test……..*”   1. Describe any methods used to examine subgroups and interactions   *-Not applicable*   1. Explain how missing data were addressed   *-Not applicable*   1. If applicable, describe analytical methods taking account of sampling strategy   *- Not applicable*   1. Describe any sensitivity analyses   *- Not applicable* |
| **Results** | | |
| **Participants** | **13** | 1. Report numbers of individuals at each stage of study—eg numbers potentially eligible, examined for eligibility, confirmed eligible, included in the study, completing follow-up, and analysed   -*result, paragraph 1 and Table 1*   1. Give reasons for non-participation at each stage   *- Not applicable*   1. Consider use of a flow diagram   - *Not applicable* |
| **Descriptive data** | **14** | 1. Give characteristics of study participants (e.g. demographic, clinical, social) and information on exposures and potential confounders   *- results, paragraph 1 and Table 1 &*  *-prevalence of podoconiois section, the whole paragraph*   1. Indicate number of participants with missing data for each variable of interest   -*results, paragarph 1 and the whole section of prevalence of podoconiois* |
| **Outcome data** | **15** | Report numbers of outcome events or summary measures  -*table 2* |
| **Main results** | **16** | 1. Give unadjusted estimates and, if applicable, confounder-adjusted estimates and their precision (eg, 95% confidence interval). Make clear which confounders were adjusted for and why they were included   - *result section and table 2*  *“.…..multivariable logistic regression analysis, age of respondents, frequency shoe wearing and their legs washing practice were significantly associated with podoconiosis. As participants age of shoe wearing delays by five years, the risk of developing podoconosis increases by 49% (AOR=1.08, 95% CI= (1.06-1.11)). Podoconosis among participants who wash their feet only by water were about 4 times higher as compared to who washed by water and soap (AOR=3.68, 95%CI= 1.47-9.24, p=0.005). The odds of developing podoconosis among those who doesn’t wear shoe daily were nearly 9 times higher compared to who wear daily (AOR=9.32, 95% CI=4.27-20.4, p=0.001)* *”*   1. Report category boundaries when continuous variables were categorized   *-not applicable*   1. If relevant, consider translating estimates of relative risk into absolute risk for a meaningful time period   - *Not applicable* |
| Other analyses | **17** | Report other analyses done—e.g. analyses of subgroups and interactions, and sensitivity analyses  -Not applicable |
| **Discussion** | **18** | Summarise key results with reference to study objectives  - results, paragraph 1 for the prevalence and paragraph 2 and 3 for associted factors  *“This study showed that, the prevalence of podoconosis in the community of Dano* *district was 6.3%………”* |
| **Limitations** | **19** | Discuss limitations of the study, taking into account sources of potential bias or imprecision. Discuss both direction and magnitude of any potential bias  -*the last paragraph of discussion section*  *“....there might be recall bias due to some questions were difficult to remember because of time”* |
| **Interpretation** | **20** | Give a cautious overall interpretation of results considering objectives, limitations, multiplicity of analyses, results from similar studies, and other relevant evidence  *-Conclusion section*  *“This study revealed that there was significant burden of Podoconiosis in the study area. Delay age of shoe wearing, washing practice of feet only by water and not daily wearing of shoe were found to be significantly associated with podoconosis”* |
| Generalisability | **21** | Discuss the generalisability (external validity) of the study results  -*the last sentence of conclusion section* |
| **Other information** | | |
| **Funding** | **22** | Give the source of funding and the role of the funders for the present study and, if applicable, for the original study on which the present article is based  *-Funding statement* |
