# Supplementary material for: Community based cross sectional study of podoconiosis and associated factors in Dano district, Central Ethiopia
Source: PLoS Negl Trop Dis. 2019 Jan 28;13(1):e0007050. doi: 10.1371/journal.pntd.0007050 (PMC6366781; doi:10.1371/journal.pntd.0007050)
Supplement: S2 Text — (DOCX) [file pntd.0007050.s002.docx]

**Questionnaire**

Identification no _________________

Date ____________

Interviewer name_________________

House hold code________

| Section 1 socio economic and Demographic factor | | | |
| --- | --- | --- | --- |
| No | Questions | Response and coding categories | skip |
| 101 | Sex | Male ……………1  Female ………….2 |  |
| 102 | Age | _____ in years |  |
| 103 | Religion | Orthodox ……….1  Protestant…………2  Muslim…………..3  Catholic………….4  Others……………5 |  |
| 104 | Ethnic group | Oromo …………..1  Amhara…………..2  Tigre ……………..3  Gurage……………4  Others,specify…….5 |  |
| 105 | What is your occupation currently? | Government employed ……….1  Nongovernmental employed….2  Merchant……………………...3  Daily laborer …………………4  Farmer ………………………..5  Student ……………………….6  Unemployed…………………..7  House wife ……………………8  Others,specify …………………9 |  |
| 106 | What is your level of education? | No education……….…………1  Able to read and write…………2  primary(1-8)…………..…….…3  secondary(9-12)…..….………..4  More than secondary….………5 |  |
| 107 | What is your monthly income? | __________ |  |
| 108 | What is your current marital status? | Never married ………………..1  Married…………………….….2  Living together………………..3  Divorced………………………4  Widowed………………………5 |  |
| 109 | What type of floor does your house have? | Mud /earth……………………..1  Wood…………………………..2  Cement……………………...…3  Other,specify……………….….4 | Observation |
| Section 2 shoe wearing and practice | | | |
| 201 | Have you ever wear shoes? | Yes……………………….……1  No……………………………..0 | Go to  207 |
| 202 | How old were you when first got your shoes? | __________in years |  |
| 203 | Is the person wearing shoes a time of interview? | Yes ………………………..….1  No ……………………………0 | Observation |
| 204 | Where do you wear shoes?  (Multiple answer possible) | At home ……………….……..1  On the field………………..….2  Other,specify………..………...3 |  |
| 205 | When do you wear shoes?  (Multiple answer possible) | During rainy season…………..1  On market days ………………2  On Sundays …………………..3  When walking far………….….4  Other,specify……………..…...5 |  |
| 206 | Frequency of wearing shoes | Daily………………………..…1  Not daily…………………..…..2 |  |
| 207 | What is cleanliness of the feet at time of interview? | Clean and intact ………………1  Dirty…………………………...2  Cracked………………………..3  Dirty and cracked……..………4 |  |
| 208 | How long does it take you to go the nearest water source? | _________ minute |  |
| 209 | When do you wash your feet? | Whenever they are dirty …..….1  Before sleeping…………..……2  Before prayer……………….…3  Other specify…………………4 |  |
| 210 | How do you wash your feet ? | By water only …. .…………....1  By water and soap………...…..2 |  |
| 211 | Frequency of feet washing? | ___________ |  |
| 212 | Do you regularly travel by bare foot for different social purpose? | Yes ………………………..….1  No……………………………2 |  |
| 213 | During farming do you wear shoe? | Yes…………………………..1  No……………………………2 | Go to 301 |
| 214 | How much time (in hour) do you spent on farming by barefoot? | ______ |  |
| Section 3 leg swelling history, ICT card results and physical examination | | | |
| 301 | Do you have leg swelling? | Yes …….1(verify by observation)  No …….0 | End the interview |
| 302 | Do you have any family member with history of leg swelling? | Yes …….1  No …….0 | Go to 304 |
| 303 | How many people in your family have leg swelling? | _______- |  |
| 304 | Where did the swelling start from? | From the foot or lower leg….….1  From high up…………………..2  Don’t know…………..………...3 |  |
| 305 | Does the swelling is on both legs ? | Yes…………………………….1  No……………………..………0 |  |
| 306 | Do you have swelling in the groin area? | Yes…………………………….1  No……………………………..0 |  |
| 307 | ICT result | Positive………………………..1  Negative………………………2 |  |
| 308 | Podoconiosis disease | Yes…………………………….1  No…………………………..…2 | End the interview |
| 309 | Does the swelling is present when you wake up first in the morning? | Yes…………………...……….1  No………………….…………0 |  |
| 310 | Does the swelling below the knee? | Yes…………………..…….….1  No…………………………….0 |  |
| 311 | Does knobs/ bumbs present above the ankle? | Yes…………………………....1  No………………………….…0 |  |
| 312 | Does the swelling is above the knee? | Yes…………………..………..1  No…………………………….0 |  |
| 313 | Does the ankle and leg are difficult to flex? | Yes…………………….……...1  No…………………………….0 |  |
| 314 | Podoconiosis disease stages | Stage 1………………………..1  Stage 2……………………..…2  Stage 3………………………..3  Stage 4………………………..4  Stage 5………………………..5 |  |
